# Non-Classic Cornelia de Lange Syndrome Due to *BRD4* Gene Alterations: A Literature Review

**DOI:** 10.3390/children12111440

**Published:** 2025-10-24

**Authors:** Fortunato Lonardo, Mariateresa Falco, Claudia Costabile, Paolo Fontana

**Affiliations:** 1 Medical Genetics Unit, P.O. Gaetano Rummo, A.O.R.N. San Pio, 82100 Benevento, Italy; mariateresa.falco@aornsanpio.it (M.F.); paolo.fontana@aornsanpio.it (P.F.); 2 AOU “L. Vanvitelli”, Dipartimento di Salute Mentale e Fisica e Medicina Preventiva, Servizio di Audiologia e Foniatria, 80138 Napoli, Italy; claudia.costabile@unicampania.it

**Keywords:** Cornelia de Lange Syndrome, CDLS6, *BRD4*, cohesin complex, growth delay, limb abnormalities, gastro-oesophageal reflux, intellectual disability, psychiatric disorder, epilepsy

## Abstract

**Highlights:**

**What are the main findings?**

- Review of the literature on the main clinical manifestations and genetic characteristics of Cornelia de Lange syndrome.
- Review of the literature on Cornelia de Lange syndrome caused by pathogenic variations in the *BRD4* gene sequence.

**What are the implications of the main findings?**

- Improve paediatricians’ knowledge of the clinical spectrum and molecular basis of this condition.
- Provide valuable information for diagnosis.

**Abstract:**

Cornelia de Lange Syndrome (CdLS) is a rare congenital disorder characterised by distinctive facial features, growth retardation, limb abnormalities and developmental delays. It is characterised by genetic heterogeneity and also presents a broad clinical variability, with a spectrum of manifestations ranging from mild to severe, with milder phenotypes that can be difficult to ascertain based on physical characteristics. Pathogenic variations in the *NIPBL* gene account for the majority of cases, but variations in several other genes, including *BRD4*, have been identified as causative factors for non-classic or milder forms of the syndrome. This review aims to analyse the roles that *BRD4* plays in the various pathways in which it is involved and to summarise current knowledge on atypical CdLS associated with *BRD4* gene alterations, highlighting clinical features, molecular mechanisms, and implications for diagnostic assessment and patient care.

## 1. Introduction

Cornelia de Lange Syndrome (CdLS) is a rare congenital disorder with an estimated prevalence of 1 in 10,000 to 30,000 live births [1]. Various forms are distinguished and listed in the Online Mendelian Inheritance in Man (OMIM) catalogue with different numbers (122470, 300590, 300882, 610759, 614701, 620568) [2].

The first report of CdLS was made in 1849 by the Dutch anatomist and pathologist Willem Vrolik [3], who described a case with a severe form of oligodactyly. In 1916, Dr Winfried Robert Clemens Brachmann reported a case characterised by bilateral monodactyly, antecubital pterygium, short stature, cervical supernumerary ribs, and increased body hair growth [4]. However, the diagnosis was formally characterised by the Dutch paediatrician Dr Cornelia de Lange, who described three unrelated cases in 1933 [5,6]. Over time, the syndrome has also been referred to by several alternative names, including Amsterdam dwarfism, Bushy syndrome, Brachmann syndrome, and Brachmann-de Lange Syndrome [7].

Pathogenic variations in the *NIPBL* gene account for the majority of cases. NIPBL is part of the cohesin protein complex, a key regulator of mitosis necessary for sister chromatid separation [8,9]. NIPBL is involved in loading the cohesin complex onto DNA, a fundamental process required for cohesin-mediated loop extrusion and the formation of Topologically Associating Domains (TADs) [10,11].

Within human cells, thousands of kilobases of DNA must be organised in a compact yet functional manner to fit into the limited space of the nucleus. This complex and dynamic organisation is primarily determined by the Structural Maintenance of Chromosomes (SMC) complexes. This family of complexes is evolutionarily conserved in both prokaryotes and eukaryotes. In eukaryotes, it includes condensin and cohesin complexes. Both complexes were initially identified as essential factors for accurate chromosome segregation, involved in mitotic/meiotic chromosome condensation and sister chromatid cohesion, respectively. Studies conducted over the past few decades have demonstrated that, in addition to their classically defined roles in mitosis, both complexes participate in several chromatin-associated processes. However, their precise mechanisms of action have not been fully elucidated yet [11]. Pathogenic variants in this complex have been associated with a growing number of syndromes, collectively known as “cohesinopathies”, the most classic being CdLS. However, even though certain overlaps exist, the clinical spectrum of cohesinopathies is very broad [12]. Indeed, the cohesin complex is involved in multiple fundamental cellular processes, including the preservation of genomic stability, regulation of transcription, modulation of chromatin architecture, and higher-order genome organisation. Given the diversity of nuclear processes that depend on a proper cohesin function, the genetic link between CdLS and the cohesin complex raised the question of which of these functions was more relevant for the pathology of the syndrome. Interestingly, in cells from patients with CdLS and in cells derived from mouse models, mitotic chromatid segregation and DNA replication remain unperturbed [11].

In recent decades, a non-cohesion-related function of this complex in transcriptional regulation has been well established, and CdLS pathoetiology has been recently associated with gene expression dysregulation [11]. This has led to a reconsideration of the adequacy of the initial categorisation as a “cohesinopathy”, and the term “transcriptomopathy” has been proposed as a more suitable alternative [13,14].

A transcriptomopathy refers to a disorder arising from widespread disruption of gene expression across the organism, accounting for the multisystem involvement and phenotypic variability characteristic of this syndrome. Although the precise pathogenic mechanisms underlying CdLS remain incompletely elucidated, the regulatory function of cohesin in controlling gene expression is considered to be a key determinant of its biological activity. The primary mechanisms implicated in CdLS involve alterations not only in cohesin but also in associated pathways, resulting from variants in genes encoding components of the transcriptional machinery, as well as proteins involved in epigenetic modification.

Recent studies have revealed unexpected roles for cohesin, cohesin loaders, and the Super Elongation Complex (SEC) in maintaining the enhancer complexes. The cohesin protein complex is essential for the formation of TADs and chromatin loops on interphase chromosomes. For loading onto chromosomes, cohesin requires the cohesin loader complex, which is formed by NIPBL and MAU2. In mammalian cells, cohesin localises with NIPBL at enhancers and gene promoters. Enhancer complexes are crucial for recruiting transcriptional regulators, sustaining active histone modifications, and facilitating enhancer-promoter looping [15].

Luna-Peláez et al. (2019) provided evidence for NIPBL and BRD4 cooperation in transcriptional regulation, which should contribute to explaining the observed CdLS-like phenotype associated with *BRD4* pathogenic variations [16].

Gaub et al. (2020) [17] showed that BRD4 and the Non-Specific Lethal (NSL) complex regulate an overlapping subset of target genes, indicating that their functional interplay is crucial for the transcriptional activation of these genes. The findings suggest that BRD4 and the NSL complex operate in a coordinated manner, forming a functional relay that facilitates the transition of RNA polymerase II from transcriptional initiation to elongation.

The finding of variations in *BRD4* and other transcriptional regulators (*ANKRD11*, *AFF4*, *ARID1B*, *EP300*, and *SETD5*, among others) in patients with characteristics similar to CdLS provides further evidence to support the hypothesis that CdLS is caused by defects in transcription [18,19]. However, an observation made by Olley et al. (2021) [20] suggests that we should still be cautious in drawing conclusions. These authors identified a missense sequence variation in *BRD4* associated with a Cornelia de Lange-like syndrome that reduces BRD4 binding to acetylated histones. They demonstrated that, although this mutation reduces BRD4 occupancy at enhancers, it does not impact the transcription of the pluripotency network in mouse embryonic stem cells. Instead, it delays the cell cycle, increases DNA damage signalling, and perturbs the regulation of DNA repair in mutant cells. This reveals a role for BRD4 in the choice of DNA repair pathway. Furthermore, they found evidence of a similar increase in DNA damage signalling in cells derived from individuals with NIPBL deficiency, suggesting that this is also a feature of typical Cornelia de Lange syndrome. Very recently, Hamilton et al. (2025) demonstrated the critical role of the genomic context in the recruitment of BRD4 to distinct classes of regulatory elements, suggesting that intergenic and gene body enhancers represent functionally distinct classes of elements [21].

**Figure 1:**
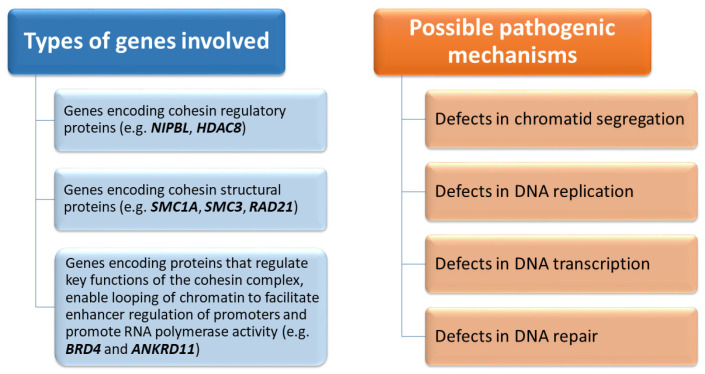
Types of genes involved in CdLS and possible pathogenic mechanisms. The names of the genes are in bold italics.

Figure 1 illustrates the categories of genes involved in the various forms of CdLS and the potential pathogenic mechanisms.

## 2. Clinical Features

### 2.1. Clinical Features of Classic CdLS

Classic CdLS exhibits a specific clinical presentation, including distinctive facial dysmorphism, prenatal and postnatal growth delay, limb abnormalities (predominantly affecting the upper limbs), excessive hair growth, and cognitive and motor delays/intellectual disability. Other clinical signs are commonly present: gastro-oesophageal reflux (GER), which is the most common and serious medical complication in patients with CdLS, sensorineural deafness, congenital abnormalities of the heart, and defects of the renal and genital tracts. This condition is generally recognisable at birth [22,23], but it may already be suspected antenatally.

The main findings detected by ultrasound during the prenatal period are increased nuchal translucency in the first trimester [24,25,26]; growth failure, which typically presents in the second trimester; and the typical in utero facial profile of a foetus with CdLS, consisting of micrognathia, a prominent upper lip, and a depressed nasal bridge with somewhat anteverted nares [26,27,28,29].

Growth retardation is very frequent. Individuals present with a generalised and symmetrical form of growth underdevelopment, leading to persistent low body weight and short stature into adulthood [30,31].

The characteristic craniofacial aspects include microcephaly, brachycephaly, synophrys, thick and arched eyebrows, long eyelashes, a short nose with a depressed nasal bridge and upturned nostrils, a long philtrum, a thin upper lip, and retrognathia [22,23,32].

Most individuals with the classic form of CdLS exhibit marked psychomotor delay and/or significant intellectual disability [33]. There is a frequent association with absent or delayed expressive communication, autism spectrum disorder, and additional behavioural difficulties. Patients may present with various neurological complications, such as seizures, motor impairments (including stereotyped movements), and abnormalities in sensory processing (like reduced pain sensitivity or thermal intolerance), muscle hypotonia, or sleep pattern anomalies. Recent evidence has demonstrated involvement of the autonomic nervous system, specifically small fibre neuropathy, which in some individuals is linked to asymmetrical sympathetic responses in the lower limbs [34]. Limb abnormalities are documented in 80% of cases. Of these, a significant proportion (25–30%) involve severe malformations, most frequently as reduction defects affecting the upper limbs, such as monodactyly or oligodactyly. Individuals without severe limb malformations usually have small hands or feet. They may also exhibit minor defects, including a proximally placed thumb or an inward curvature of the fingers (clinodactyly). Such anomalies are generally bilateral but present asymmetrically [35]. GER affects the vast majority of patients (93.3%) [36,37]. Given the high prevalence and potential link to feeding difficulties, a clinical assessment for GER should be conducted early, utilising oesophageal pH impedance or manometry. When these methods are unavailable, a barium swallow can be used as an alternative. Inadequate management of GER can result in various complications, including oesophagitis, pneumonitis/aspiration pneumonia, and behavioural issues (such as irritability or self-injurious behaviour). These behavioural symptoms are often addressed inappropriately with psychotropic drugs. Less frequently observed gastrointestinal abnormalities, such as intestinal malrotation (reported in 2–3% of cases), also require careful consideration. Specifically, this should always be ruled out in CdLS patients who present at the emergency department with acute abdominal pain.

Furthermore, pyloric stenosis (found in 7% of cases) or congenital diaphragmatic hernia may also occur [38]. The most frequent sensory complication is sensorineural hearing loss, affecting 80% of patients. Of these, nearly half experience severe bilateral hearing loss [39]. Regarding vision, palpebral ptosis, typically bilateral, and myopia (present in more than 50% of cases) are common, as is nystagmus (occurring in approximately 40% of cases). Less common ophthalmological manifestations comprise nasolacrimal duct obstruction, strabismus, microcornea, glaucoma, and optic nerve coloboma [40]. Regarding the cardiovascular system, approximately one-third of patients have congenital heart anomalies, most frequently atrial or ventricular septal defect, pulmonary valve stenosis, tetralogy of Fallot, hypoplastic left heart syndrome and bicuspid aortic valve [41]. A recent study demonstrated that some patients may develop early cardiomyopathy, which can be detected through speckle-tracking echocardiography before the onset of symptoms or other echocardiographic or laboratory abnormalities [42]. In the genitourinary system, cryptorchidism is frequent in male patients (75%), bicornate uterus in female patients (25%), and hypoplasia of genitalia in patients of any sex (50%). Renal anomalies are rare, and the most frequent one is vesicoureteral reflux (10%) [43]. A degree of endocrine dysregulation has also been described, including abnormal levels of prolactin and delayed puberty. Abnormal results in the Homeostatic Model Assessment for Insulin Resistance (HOMA-IR) have been associated with early development of insulin resistance in some patients [44]. Low levels of lean mass and decreased bone density have also been reported [45]. Other, less frequently identified abnormalities include cleft palate, dental anomalies, cutis marmorata and low-pitched cry [22].

### 2.2. Clinical Features of Non-Classic CdLS

One of the most significant diagnostic challenges in CdLS is determining the prevalence of mutation-negative individuals who harbour undetected mosaic mutations in known CdLS genes [46]. The presence of mosaicism, in fact, can modify the classic phenotype, making the clinical characteristics less striking.

In addition to cases involving mosaicism, advances in genetic diagnosis have made it possible to identify additional, usually milder, non-classic phenotypes of CdLS that can be difficult for paediatricians to identify.

The significant clinical variability observed in atypical cases has led some authors to adopt the terminology ‘Cornelia de Lange spectrum’ in place of syndrome. Compared to the classic form, atypical patients tend to have less distinct characteristics. Their main identifying features may be synophrys and the facial gestalt [47].

### 2.3. Clinical Features of Non-Classic CdLS Due to BRD4 Mutations

Patients with CdLS who have a pathogenic variant in the *BRD4* gene are extremely rare. They represent less than 1% of the patients with CdLS. There are very few cases described in the literature; therefore, it is not yet possible to establish a comprehensive clinical profile. Despite this, some characteristics have emerged that differentiate these patients from those with the classic form of CdLS.

Olley et al. (2018) [48] investigated a cohort of 92 individuals exhibiting clinical features consistent with Cornelia de Lange syndrome but lacking variants in the known causative genes and identified four unrelated patients with microcephaly, developmental delay, impaired intellectual development (ID), and dysmorphic facial features, including synophrys, arched eyebrows, a short nose, and a long philtrum. One patient had a typical facial appearance of CdLS, two patients were classified as having atypical CdLS based on facial features, and one patient had developmental delay, ventricular septal defect, cleft lip, and hypertelorism but was not suspected of having CdLS. All four patients had a mutation in the *BRD4* gene. Two further individuals, not included in the initial cohort, were subsequently found to carry pathogenic variants in the BRD4 gene. The authors also re-examined the phenotypic profiles of additional patients harbouring heterozygous multigene deletions encompassing *BRD4*. They recognised a significant overlap with the CdLS phenotype, suggesting that *BRD4* haploinsufficiency is the likely cause of an atypical form of CdLS referred to as Cornelia de Lange Syndrome 6 (CDLS6).

Through an international collaboration, Jouret et al. (2022) [49] described 12 patients, ranging in age from 10 weeks to 32 years, and two prenatal cases with microcephaly, intrauterine growth restriction, and other features. Microcephaly was present in 12 of the 14 patients. Initial global developmental delay was seen in 12 patients. Impaired intellectual development was identified in five of 11 patients over the age of three years, four of 11 had learning difficulties without impaired ID, and two had an IQ within the normal range without learning difficulties. Five of 11 patients had psychiatric disorders, including psychotic disorder, schizophrenia, obsessive–compulsive disorder, poor performance in socialisation, and other conditions. A recognisable pattern of facial features, including arched eyebrows, sometimes with synophrys, a frontal upsweep of hair, prominent incisors, and a short nose with anteverted nostrils, was observed in six of the seven patients. The authors noted that the phenotype evolved with age and that none of the patients exhibited a classic CdLS phenotype, as none had growth failure, hypertrichosis, or radial and limb anomalies.

From a general perspective, patients with *BRD4* pathogenic variations often exhibit milder or atypical features compared to those with classic CdLS. Common characteristics include mild facial dysmorphisms, such as a high forehead, thin, arched eyebrows, and a short nose, along with variable developmental delays. Most individuals may lack the characteristic limb defects seen in classic cases, making diagnosis more challenging.

Table 1 presents a comparison of the principal clinical characteristics of individuals with CdLS arising from pathogenic variants in the major causative genes.

## 3. Molecular Findings and Genetic Testing

Several genes located on distinct chromosomes are involved in the formation of the cohesin complex, and a total of seven genes (five autosomal and two X-linked) have been linked to CdLS [22,50,51,52,53,54]. Five principal genes are responsible for roughly 70% of the reported cases [8,54]. Among these five genes, *NIPBL*, located on chromosome 5, is responsible for approximately 60% of cases, while the other genes (SMC1A, HDAC8, SMC3, and RAD21) collectively account for the remaining 10% of cases [22,55].

**Figure 2:**
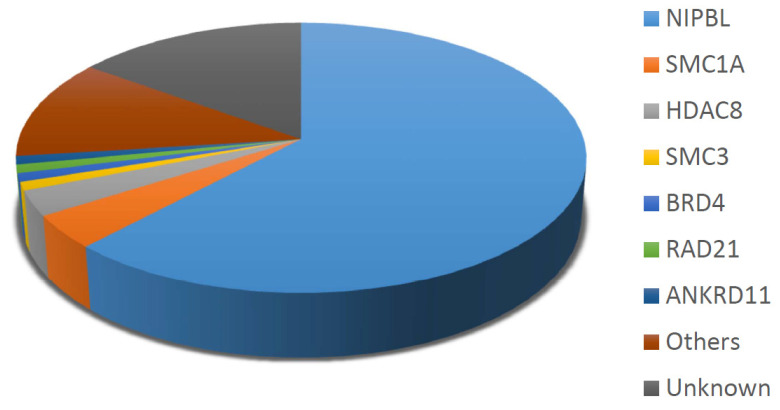
Estimated prevalence of CdLS cases linked to pathogenic variants within a specific gene. Data from [1,53].

However, advances in genetic testing have uncovered additional genetic contributors, including *BRD4*, which is associated with atypical or milder phenotypes of CdLS. Rare Copy Number Variants (CNVs) involving 1p36.23–36.22, 7p22.3, 17q24.2–25.3, 19p13.3 and 20q11.2-q12 have also been reported in association with CdLS-like features [56]. Figure 2 illustrates the distribution of sequence variations in various genes among patients with CdLS.

For individuals presenting with the classic CdLS phenotype, the recommended first-line molecular diagnostic strategy is Next-Generation Sequencing (NGS)–based analysis, such as Whole-Exome Sequencing (WES) or Whole-Genome Sequencing (WGS), with particular attention to the *NIPBL*, *SMC1A*, *SMC3*, *RAD21*, *BRD4*, *HDAC8*, and *ANKRD11* genes. This approach is recommended instead of simply sequencing the *NIPBL* gene. In cases displaying an atypical CdLS phenotype, genetic testing by WES or WGS may be considered on an individual basis [7]. In some cases, the patient’s phenotype can guide experienced clinicians in prioritising the sequencing of specific candidate genes. When this is not possible, WES or WGS can be undertaken.

**Figure 3:**
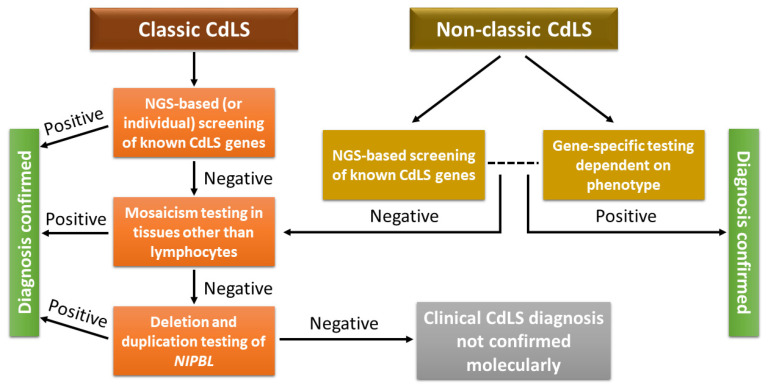
A suggested diagnostic workflow for identifying molecular causes of both classic and atypical CdLS [22]. The dotted line indicates two alternative possibilities.

The presence of somatic mosaicism in some individuals (10–13%) may pose an additional challenge, requiring analysis of other tissues, such as oral mucosa specimens, fibroblasts, or bladder epithelial cells from urine, and the use of highly sensitive molecular diagnostic techniques when results from blood samples are negative. If this mosaicism-focused approach yields a negative result, deletion and duplication analysis of the *NIPBL* gene should then be performed employing Multiplex Ligation-Dependent Probe Amplification (MLPA) or a microarray-based analytical approach [7]. Figure 3 illustrates the suggested diagnostic workflow for identifying the molecular causes of CdLS [22].

However, despite significant advancements, a substantial number of individuals still lack a definitive genetic diagnosis, indicating that other causative genes and mutational mechanisms remain to be discovered [47]. Rentas et al. (2020) [57] proposed RNA sequencing (RNA-seq) as the best approach available to detect genome-wide differences in transcript abundance and splicing, with the added advantage of being able to identify germline exonic single-nucleotide variants (SNVs), indels, and allele-specific expression. They demonstrated the utility of RNA-seq on patient-derived B-lymphoblastoid cell lines (LCLs) from patients with a clinical diagnosis of CdLS, also supporting that LCLs are superior to blood for diagnostic testing.

The use of advanced tools such as optical genome mapping (OGM), linked-read WGS, and long-read sequencing (LRS) is not recommended as a routine procedure. However, it may be helpful in specific cases that cannot be resolved using other techniques. In a patient described by Bestetti et al. (2024) [58], for example, the clinical picture was highly suggestive of CdLS. However, all the tests performed, including karyotyping, *NIPBL* gene sequencing, MLPA, a-CGH, and fluorescent in situ hybridisation (FISH), revealed an abnormal karyotype but failed to explain the clinical picture. RT-qPCR revealed the expression of a truncated transcript, likely resulting in a defective protein, which confirmed the CdLS diagnosis; however, the molecular mechanism underlying this event remained an unresolved challenge for years. The LRS approach, utilising nanopore technologies, filled the gap and highlighted a chromothripsis event. Sixteen breaks disrupted the NIPBL gene, and the resulting fragments were relocated in different positions and orientations.

Dey et al. (2000) [59] successfully cloned the murine Brd4 gene, which they designated as Mcap. The encoded protein comprises two N-terminal bromodomains, a central extraterminal (ET) domain, and several kinase-like motifs within its N-terminal region. Through database analysis, the authors also identified a human cDNA encoding a 731-amino-acid protein that mirrors the domain architecture of the N-terminal portion of murine Brd4 but lacks its C-terminal region. Indirect immunofluorescence assays performed on HeLa cells and two murine cell lines revealed nuclear localisation of BRD4. During interphase, BRD4 showed a finely granular distribution throughout the nucleus, with exclusion from the nucleoli. In mitotic cells, the distribution of BRD4 was confined almost entirely to condensed chromosomes.

French et al. (2001) [60] identified two alternatively spliced isoforms of BRD4 that produce proteins identical in their N-terminal regions, encompassing two bromodomains, an extraterminal (ET) domain, and a serine-rich segment. The longer isoform possesses an additional C-terminal extension characterised by proline- and glutamine-rich regions. The authors further demonstrated that the BRD4 coding sequence comprises 19 exons and, through analysis of a translocation involving chromosome 19, localised the *BRD4* gene to chromosomal region 19p13.1.

You et al. (2004) [61] identified the full-length cDNA for BRD4 through a two-step process involving the screening of an expressed sequence tag (EST) database and PCR amplification from a human cDNA library. The resulting BRD4 protein contains 1362 amino acids.

Using Northern blot analysis, French et al. (2003) demonstrated the widespread expression of BRD4, detecting 4.4 kb and 6.0 kb transcripts, which are presumed to encode the short and long BRD4 isoforms, respectively [62].

**Figure 4:**
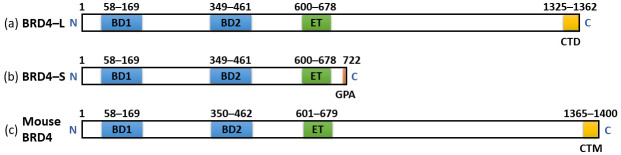
Structure of BRD4 isoforms showing the locations of various domains. The numbers indicate the amino acid residues encompassing the domain. (**a**) Human BRD4 long isoform (BRD4-L) is a protein of approximately 200 kDa that contains two tandem bromodomains (BD1 and BD2), one extra-terminal domain (ET) and a C-terminal domain (CTD). (**b**) Human BRD4 short isoform (BRD4-S) is a protein of approximately 120 kDa that contains two tandem bromodomains (BD1 and BD2), one extra-terminal domain (ET) and a terminal domain composed of three amino acids: glycine (G), proline (P) and alanine (A). (**c**) Mouse BRD4 is a protein of approximately 200 kDa that contains two tandem bromodomains (BD1 and BD2), one extra-terminal domain (ET) and a conserved C-terminal motif (CTM). Modified and adapted from [63,64,65].

Figure 4 illustrates the structure of the two isoforms of human BRD4 and the most common isoform of murine BRD4.

BRD4 is a member of the bromodomain and extraterminal domain (BET) protein family with tandem bromodomains that ‘read’ acetylated lysine marks on chromatin. It binds mainly to hyperacetylated genomic regions, which encompass promoters and enhancers, and BRD4 levels are particularly high at super-enhancers [20,66]. Super-enhancers are clustered cis-regulatory elements (CREs) that control genes important for cell type specification. Super-enhancers are molecularly defined as genomic intervals with high levels of H3K27 acetylation and binding of both BRD4 and the mediator complex [67]. A critical role for cohesin in the function of super-enhancers has been recently reported [68]. Acute depletion of cohesin led to the disruption of higher-order chromatin structure and disordered transcription of genes predicted to be under the control of super-enhancers.

Pathogenic variations in *BRD4* have been linked to a spectrum of developmental disorders, including a subset of cases presenting with features overlapping with CdLS.

Olley et al. (2018) [48], among 92 patients with features of Cornelia de Lange syndrome who were negative for mutations in known causative genes, reported four unrelated patients with mutations in the *BRD4* gene. Two patients had de novo heterozygous mutations: a missense mutation and a 1.04 Mb deletion that included *BRD4* and 28 other protein-coding genes. Two additional patients, who were not part of the original cohort, were identified as having de novo heterozygous frameshift mutations in the *BRD4* gene. The authors then reviewed the phenotypes of other patients with heterozygous multigene deletions that encompass the *BRD4* gene. They recognised a significant overlap with the CdLS phenotype, suggesting that BRD4 haploinsufficiency is the likely cause of an atypical form of CdLS referred to as Cornelia de Lange Syndrome 6 (CDLS6). The authors showed that the *BRD4* missense variant resulted in a more typical presentation of CdLS; this variant retained the ability to coimmunoprecipitate with NIPBL but exhibited decreased binding to acetylated histones of promoter and super-enhancer genes. Functional analyses demonstrated that BRD4 and NIPBL coregulated binding at super-enhancer genes and appeared to coregulate developmental gene expression.

Jouret et al. (2022) [49] described other 14 patients. The mutations included eight point mutations and six large deletions. Of the eight point mutations, four were premature truncating variations and four were missense variations. Deletion size varied from 46 kb to 2.2 Mb; the 46 kb deletion overlapped only the *BRD4* gene.

The heterozygous mutations in the *BRD4* gene that were identified in patients with CDLS6 by Olley et al. (2018) [48] and Jouret et al. (2022) [49] occurred de novo [69].

## 4. Discussion

CDLS6 is a distinct, atypical form of CdLS resulting from pathogenic variations in *BRD4* and is listed in the OMIM catalogue under number #620568 [69]. It has an autosomal dominant transmission.

The *BRD4* gene encodes a ubiquitously expressed chromatin-binding protein that belongs to the BET family. Structurally, BRD4 protein contains two bromodomains, an extraterminal (ET) domain, and a C-terminal motif (CTM) (Figure 4), each mediating specific protein–protein interactions. BRD4, like other BET proteins, acts as an epigenetic reader, recognising acetylated lysine residues on histone tails BD1 and BD2. Through this recognition, BRD4 serves as a scaffold linking acetylated chromatin regions to the transcriptional machinery, thereby coordinating chromatin remodelling and transcriptional elongation.

Beyond transcriptional control, BRD4 is implicated in multiple nuclear processes, including DNA damage repair, cell cycle progression, and maintenance of chromosomal integrity. Therefore, it is not surprising that alterations in BRD4 activity or expression contribute to a broad spectrum of human disorders.

Gain-of-function mutations and chromosomal translocations can cause or promote various types of cancer. BRD4 is important in tumorigenesis largely due to its involvement in super-enhancer (SE) organisation and the regulation of oncogene expression, especially c-MYC, which is well established to be highly dependent on BRD4 activity [70].

This pivotal role has stimulated substantial interest in the identification, rational design, and development of BRD4 inhibitors (BRD4i), a promising class of small-molecule agents that are presently progressing through multiple stages of clinical evaluation [71].

Conversely, loss-of-function or haploinsufficient variants in BRD4 have been identified in patients with Cornelia de Lange syndrome type 6 (CDLS6) [69].

Evidence of cooperation between NIPBL and BRD4 in regulating transcription may help explain the partial overlap between the phenotypes caused by NIPBL alterations and those caused by BRD4 alterations [16]. Functional studies suggest that BRD4 deficiency disrupts transcriptional elongation and enhancer–promoter interactions, which are crucial for normal morphogenesis [19]. Consequently, alterations in BRD4 that disrupt its regulatory control over cohesin-associated genes and developmental transcriptional pathways result in pre- and postnatal growth deficiency, craniofacial dysmorphism, limb malformations, and neurodevelopmental impairment.

To our knowledge, fewer than 20 patients are described in the literature. The subjects reported share the classic CdLS phenotype’s developmental delay, microcephaly, and some of its facial features, including arched eyebrows, synophrys, a small nose with anteverted nares, and dental anomalies. On the other hand, they usually show normal growth, do not have limb defects or hypertrichosis, and have a milder intellectual disability (identified only in about half of the cases). No correlation was identified between the severity of neurodevelopmental delay and the type of variant. However, patients with a normal IQ also usually present with learning difficulties [49,72].

A high incidence of psychiatric disorders has been observed in patients with *BRD4* loss-of-function variants. In cases reported in the literature, various disorders have been described, including psychotic disorder, schizophrenia, obsessive–compulsive disorder, and dissociative identity disorder, suggesting that psychiatric disorders could be a peculiar feature of this subgroup of CdLS. A patient who came to our observation at the age of 13 shows mood disorder and anxiety disorder, but his phenotype could evolve in the following years, and the psychiatric follow-up has to be very meticulous.

None of the patients reported has the classic phenotype of CdLS: in particular, short stature, hypertrichosis, and limb defects are usually absent. The facial features, including arched eyebrows, synophrys, a small nose with anteverted nares, and micrognathy, are distinctive and represent the main diagnostic handle that clinicians can use to suspect a CdLS spectrum, despite the absence of key features such as growth deficiency and hypertrichosis. Microcephaly has been reported in the majority of cases. Several individuals have seizures. It will be necessary to collect a larger number of cases in order to assess the prevalence of epilepsy and compare it with the known prevalence of epilepsy in CdLS, which is estimated at 25%.

## 5. Conclusions

Although the classic form of Cornelia de Lange syndrome (CdLS) was formally described over ninety years ago and is now well characterised from a clinical standpoint [73,74], the identification of the molecular genetic basis of CdLS has led to the recognition of individuals with milder or atypical features. Therefore, this condition encompasses a spectrum of findings from mild to severe. The less clinically recognisable, milder form of CdLS may account for the majority of affected individuals [75,76]. Central to making the diagnosis is the identification of diagnostic facial features. Important aspects include eyebrows, nasal features, a thin upper lip, and micrognathia. However, it is essential to remember that, like other syndromes, CdLS is most accurately diagnosed in childhood, and the diagnosis becomes increasingly difficult with age [76].

Pathogenic variations in *BRD4* represent an important genetic cause of atypical CdLS, characterised by a clinically relevant and recognisable phenotype, distinguishable from the other cohesinopathies and especially different from the classic CdLS, thereby contributing to the expanding understanding of the syndrome’s clinical spectrum and molecular basis.

In several syndromes, the initial phenotype during infancy may be suggestive of the CdLS-like spectrum and then evolve into a different core phenotype. Long-term follow-up of patients and the identification of new adult cases will enable us to determine whether this also applies to CDLS6 and to further characterise its core phenotype.

This study enables a preliminary clinical delineation of this syndrome, which, despite being described in a very limited number of patients to date, is likely underdiagnosed and appears to have remarkable clinical expressivity. Future descriptions of new patients and their molecular data will be valuable for elucidating the full phenotypic spectrum, investigating potential genotype–phenotype correlations, and developing targeted therapies.

The phenotypic variability underscores the importance of comprehensive genetic testing in cases where a diagnosis is suspected. However, despite the identification of new genes related to rare cases of CdLS, several patients are still diagnosed with idiopathic CdLS, suggesting that other molecular mechanisms of this condition remain to be discovered.

## Figures and Tables

**Table 1:** Comparison of the key clinical features observed in individuals with molecularly confirmed CdLS resulting from pathogenic variants in the major disease-associated genes [22,49]. The key clinical features, also indicated by their HPO ID, are grouped into categories. The categories are indicated in bold italics and highlighted in grey. The column relating to the *BRD4* gene is highlighted in light grey.

	HPO ID	*NIPBL*	*SMC1A*	*SMC3*	*BRD4*	*HDAC8*	*RAD21*	*ANKRD11*
***Growth***								
IUGR	0001511	+++	++	+	++/+	++	++	−
Short stature	0004322	+++	++	++	+	+	++	++
Microcephaly	0000252	++++	++	++	++/+++	+	++	+
***Craniofacial features***								
Brachycephaly	0000248	++	+	+++	+	+++	++	+
Low anterior hairline	0000294	+++	+++	+++	++/+	++	+	+
Arched, thick eyebrows	0002253, 0000574	+++	+++	++++	+++/++++	+++	+++	+
Synophrys	0000664	++++	+++	+++	+++/++	++++	+++	+
Long eyelashes	0000527	++++	+++	+++	+	+	+++	+
Depressed nasal bridge	0005280	+++	+	+	+	+	+	− ^a^
Anteverted nostrils	0000463	+++	++	++	++/+++	+++	+++	+
Broad nasal tip	0000455	++	++	+++	+	+	−	++
Long, smooth philtrum	0000343, 0000319	+++	++	++	++	++	++	++
Thin upper vermilion	0000219	++++	+++	+++	++	+	+++	++
Downturned corners of the mouth	0002714	++++	+++	++	+	++	+++	−
Highly arched palate	0000218	++	+	+	+	+	++	+
Widely spaced teeth	0000687	+++	+	+	−	++	−	− ^b^
Micrognathia	0000347	+++	+	+	++	++	+	−
Low-set and malformed ears	0000369, 0000377	++	+	+	−	+	+	−
***Trunk and limbs***								
Oligodactyly and adactyly (hands)	0012165, 0009776	+	−	−	−	−	−	−
Small hands	0200055	+++	+++	+++	++	++++	+++	++
Proximally placed thumbs	0009623	++	+	+++	+++	+++	+	−
Clinodactyly or short fifth finger	0004209, 0009237	+++	+	++	+	++	+++	++
Small feet	0001773	++++	++	+++	NR	+++	+++	+
Hirsutism	0001007	+++	+++	++++	−	+	++	++
Cardiovascular anomalies	0002564	+	+	+	+	+	+	−
Vertebral anomalies	0003468	−	−	+	−	−	++	+++
***Cognition and behaviour***								
Intellectual disability (any degree)	0001249	++++	++++	++++	++++	++++	+	++++
ASD	0000729	+	+	+	−	+	+	+
Self-injurious behaviour	0100716	+++	+	NR	+	+	−	++
Stereotypic movements	0000733	++	++	NR	NR	−	−	−

ASD, autism spectrum disorder; HPO ID, Human Phenotype Ontology identifier; IUGR, intrauterine growth retardation; NR, not reported; ++++, ≥90%; +++, 70–89%; ++, 50–69%; +, 20–49%; −, <20%. ^a^ Prominent nasal bridge. ^b^ Macrodontia (larger than normal teeth).

## Data Availability

No new data were created or analyzed in this study. Data sharing is not applicable to this article.

## Abbreviations

The following abbreviations are used in this manuscript:

CdLS	Cornelia de Lange Syndrome
OMIM	Online Mendelian Inheritance in Man
TADs	Topologically Associating Domains
SMC	Structural Maintenance of Chromosomes
SEC	Super Elongation Complex
NSL	Non-Specific Lethal
CNVs	Copy Number Variants
NGS	Next-Generation Sequencing
WES	Whole-Exome Sequencing
WGS	Whole-Genome Sequencing
MLPA	Multiplex Ligation-Dependent Probe Amplification
RNA-seq	RNA sequencing
LCLs	Lymphoblastoid Cell Lines
BET	Bromodomain and Extra-Terminal domain
CREs	Cis-Regulatory Elements
CDLS6	Cornelia de Lange Syndrome 6
GER	Gastro-Esophageal Reflux
HOMA-IR	Homeostatic Model Assessment for Insulin Resistance

## References

[1] Kaur M., Blair J., Devkota B., Fortunato S., Clark D., Lawrence A., Kim J., Do W., Semeo B., Katz O. (2023). Genomic Analyses in Cornelia de Lange Syndrome and Related Diagnoses: Novel Candidate Genes, Genotype-Phenotype Correlations and Common Mechanisms. Am. J. Med. Genet. A.

[2] (2025). Online Mendelian Inheritance in Man, OMIM^®^.

[3] Vrolik W. (1849). Tabulae Ad Illustrandam Embryogenesin Hominis et Mammalium, Tam Naturalem Quam Abnormem.

[4] Brachmann W.R.C. (1916). Ein Fall von Symmetrischer Monodaktylie Durch Ulnadefekt, Mit Symmetrischer Flughautbildung in Den Ellenbogen, Sowie Anderen Abnormalitaten (Zwerghaftigkeit, Halsrippen, Behaarung). Jahrb. Kinderheilkd. Phys. Erzieh..

[5] de Lange C. (1933). Sur Un Type Nouveau de Degeneration (Typus Amstelodamensis) [On a New Type of Degeneration (Type Amsterdam)]. Arch. Med. Enfants.

[6] Oostra R., Baljet B., Hennekam R.C.M. (1994). Brachmann-de Lange Syndrome “Avant La Lattre”. Am. J. Med. Genet..

[7] Cascella M., Muzio M.R. (2025). Cornelia de Lange Syndrome. StatPearls.

[8] Szyca R., Leksowski K. (2011). Cornelia de Lange Syndrome—Characteristics and Laparoscopic Treatment Modalities of Reflux Based on Own Material. Videosurgery Other Miniinvasive Tech..

[9] Liu J., Krantz I.D. (2009). Cornelia de Lange Syndrome, Cohesin, and Beyond. Clin. Genet..

[10] Newkirk D.A., Chen Y.-Y., Chien R., Zeng W., Biesinger J., Flowers E., Kawauchi S., Santos R., Calof A.L., Lander A.D. (2017). The Effect of Nipped-B-like (Nipbl) Haploinsufficiency on Genome-Wide Cohesin Binding and Target Gene Expression: Modeling Cornelia de Lange Syndrome. Clin. Epigenetics.

[11] García-Gutiérrez P., García-Domínguez M. (2021). BETting on a Transcriptional Deficit as the Main Cause for Cornelia de Lange Syndrome. Front. Mol. Biosci..

[12] Piché J., Van Vliet P.P., Pucéat M., Andelfinger G. (2019). The Expanding Phenotypes of Cohesinopathies: One Ring to Rule Them All!. Cell Cycle.

[13] Yuan B., Pehlivan D., Karaca E., Patel N., Charng W.-L., Gambin T., Gonzaga-Jauregui C., Sutton V.R., Yesil G., Bozdogan S.T. (2015). Global Transcriptional Disturbances Underlie Cornelia de Lange Syndrome and Related Phenotypes. J. Clin. Investig..

[14] Kawauchi S., Santos R., Muto A., Lopez-Burks M.E., Schilling T.F., Lander A.D., Calof A.L. (2016). Using Mouse and Zebrafish Models to Understand the Etiology of Developmental Defects in Cornelia de Lange Syndrome. Am. J. Med Genet. Part C Semin. Med Genet..

[15] Sakata T., Tei S., Izumi K., Krantz I.D., Bando M., Shirahige K. (2025). A Common Molecular Mechanism Underlying Cornelia de Lange and CHOPS Syndromes. Curr. Biol..

[16] Luna-Peláez N., March-Díaz R., Ceballos-Chávez M., Guerrero-Martínez J.A., Grazioli P., García-Gutiérrez P., Vaccari T., Massa V., Reyes J.C., García-Domínguez M. (2019). The Cornelia de Lange Syndrome-Associated Factor NIPBL Interacts with BRD4 ET Domain for Transcription Control of a Common Set of Genes. Cell Death Dis..

[17] Gaub A., Sheikh B.N., Basilicata M.F., Vincent M., Nizon M., Colson C., Bird M.J., Bradner J.E., Thevenon J., Boutros M. (2020). Evolutionary Conserved NSL Complex/BRD4 Axis Controls Transcription Activation via Histone Acetylation. Nat. Commun..

[18] Izumi K., Nakato R., Zhang Z., Edmondson A.C., Noon S., Dulik M.C., Rajagopalan R., Venditti C.P., Gripp K., Samanich J. (2015). Germline Gain-of-Function Mutations in AFF4 Cause a Developmental Syndrome Functionally Linking the Super Elongation Complex and Cohesin. Nat. Genet..

[19] Parenti I., Kaiser F.J. (2021). Cornelia de Lange Syndrome as Paradigm of Chromatinopathies. Front. Neurosci..

[20] Olley G., Pradeepa M.M., Grimes G.R., Piquet S., Polo S.E., FitzPatrick D.R., Bickmore W.A., Boumendil C. (2021). Cornelia de Lange Syndrome-Associated Mutations Cause a DNA Damage Signalling and Repair Defect. Nat. Commun..

[21] Hamilton G.A., Ruiz P.D., Ye K., Gamble M.J. (2025). Acetylation of Histone H2B on Lysine 120 Regulates BRD4 Binding to Intergenic Enhancers. bioRxiv.

[22] Kline A.D., Moss J.F., Selicorni A., Bisgaard A.-M., Deardorff M.A., Gillett P.M., Ishman S.L., Kerr L.M., Levin A.V., Mulder P.A. (2018). Diagnosis and Management of Cornelia de Lange Syndrome: First International Consensus Statement. Nat. Rev. Genet..

[23] Kline A.D., Krantz I.D., Sommer A., Kliewer M., Jackson L.G., FitzPatrick D.R., Levin A.V., Selicorni A. (2007). Cornelia de Lange Syndrome: Clinical Review, Diagnostic and Scoring Systems, and Anticipatory Guidance. Am. J. Med. Genet. A.

[24] Sekimoto H., Osada H., Kimura H., Kamiyama M., Arai K., Sekiya S. (2000). Prenatal Findings in Brachmann-de Lange Syndrome. Arch. Gynecol. Obstet..

[25] Huang W.H., Porto M. (2002). Abnormal First-Trimester Fetal Nuchal Translucency and Cornelia De Lange Syndrome. Obstet. Gynecol..

[26] Clark D.M., Sherer I., Deardorff M.A., Byrne J.L.B., Loomes K.M., Nowaczyk M.J.M., Jackson L.G., Krantz I.D. (2012). Identification of a Prenatal Profile of Cornelia de Lange Syndrome (CdLS): A Review of 53 CdLS Pregnancies. Am. J. Med. Genet. A.

[27] Ranzini A.C., Day-Salvatore D., Farren-Chavez D., McLean D.A., Greco R. (1997). Prenatal Diagnosis of de Lange Syndrome. J. Ultrasound Med..

[28] Boog G., Sagot F., Winer N., David A., Nomballais M.F. (1999). Brachmann-de Lange Syndrome: A Cause of Early Symmetric Fetal Growth Delay. Eur. J. Obstet. Gynecol. Reprod. Biol..

[29] Urban M., Hartung J. (2001). Ultrasonographic and Clinical Appearance of a 22-Week-Old Fetus with Brachmann-de Lange Syndrome. Am. J. Med. Genet..

[30] Bruner J.P., Hsia Y.E. (1990). Prenatal Findings in Brachmann-de Lange Syndrome. Obstet. Gynecol..

[31] Kline A.D., Barr M., Jackson L.G. (1993). Growth Manifestations in the Brachmann-de Lange Syndrome. Am. J. Med. Genet..

[32] Mannini L., Cucco F., Quarantotti V., Krantz I.D., Musio A. (2013). Mutation Spectrum and Genotype-Phenotype Correlation in Cornelia de Lange Syndrome. Hum. Mutat..

[33] Moss J., Oliver C., Arron K., Burbidge C., Berg K. (2009). The Prevalence and Phenomenology of Repetitive Behavior in Genetic Syndromes. J. Autism Dev. Disord..

[34] Pablo M.J., Pamplona P., Haddad M., Benavente I., Latorre-Pellicer A., Arnedo M., Trujillano L., Bueno-Lozano G., Kerr L.M., Huisman S.A. (2021). High Rate of Autonomic Neuropathy in Cornelia de Lange Syndrome. Orphanet J. Rare Dis..

[35] Mehta D., Vergano S.A.S., Deardorff M., Aggarwal S., Barot A., Johnson D.M., Miller N.F., Noon S.E., Kaur M., Jackson L. (2016). Characterization of Limb Differences in Children with Cornelia de Lange Syndrome. Am. J. Med. Genet. C Semin. Med. Genet..

[36] Luzzani S., Macchini F., Valadè A., Milani D., Selicorni A. (2003). Gastroesophageal Reflux and Cornelia de Lange Syndrome: Typical and Atypical Symptoms. Am. J. Med. Genet. A.

[37] Kline A.D., Grados M., Sponseller P., Levy H.P., Blagowidow N., Schoedel C., Rampolla J., Clemens D.K., Krantz I., Kimball A. (2007). Natural History of Aging in Cornelia de Lange Syndrome. Am. J. Med. Genet. C Semin. Med. Genet..

[38] Marino T., Wheeler P.G., Simpson L.L., Craigo S.D., Bianchi D.W. (2002). Fetal Diaphragmatic Hernia and Upper Limb Anomalies Suggest Brachmann-de Lange Syndrome. Prenat. Diagn..

[39] Sataloff R.T., Spiegel J.R., Hawkshaw M., Epstein J.M., Jackson L. (1990). Cornelia de Lange Syndrome. Otolaryngologic Manifestations. Arch. Otolaryngol. Head. Neck Surg..

[40] Nallasamy S., Kherani F., Yaeger D., McCallum J., Kaur M., Devoto M., Jackson L.G., Krantz I.D., Young T.L. (2006). Ophthalmologic Findings in Cornelia de Lange Syndrome: A Genotype-Phenotype Correlation Study. Arch. Ophthalmol..

[41] Ayerza Casas A., Puisac Uriol B., Teresa Rodrigo M.E., Hernández Marcos M., Ramos Fuentes F.J., Pie Juste J. (2017). Cornelia de Lange Syndrome: Congenital Heart Disease in 149 Patients. Med. Clin..

[42] Trujillano L., Ayerza-Casas A., Puisac B., García G.G., Ascaso Á., Latorre-Pellicer A., Arnedo M., Lucia-Campos C., Gil-Salvador M., Kaiser F.J. (2022). Subclinical Myocardial Dysfunction Is Revealed by Speckle Tracking Echocardiography in Patients with Cornelia de Lange Syndrome. Int. J. Cardiovasc. Imaging.

[43] Oliver C., Bedeschi M.F., Blagowidow N., Carrico C.S., Cereda A., Fitzpatrick D.R., Gervasini C., Griffith G.M., Kline A.D., Marchisio P. (2010). Cornelia de Lange Syndrome: Extending the Physical and Psychological Phenotype. Am. J. Med. Genet. A.

[44] Ascaso Á., Latorre-Pellicer A., Puisac B., Trujillano L., Arnedo M., Parenti I., Llorente E., Puente-Lanzarote J.J., Matute-Llorente Á., Ayerza-Casas A. (2024). Endocrine Evaluation and Homeostatic Model Assessment in Patients with Cornelia de Lange Syndrome. J. Clin. Res. Pediatr. Endocrinol..

[45] Matute-Llorente Á., Ascaso Á., Latorre-Pellicer A., Puisac B., Trujillano L., Llorente E., Puente-Lanzarote J.J., Ayerza-Casas A., Arnedo M., Moreno L.A. (2021). Targeted Gene Sequencing, Bone Health, and Body Composition in Cornelia de Lange Syndrome. Appl. Sci..

[46] Ansari M., Poke G., Ferry Q., Williamson K., Aldridge R., Meynert A.M., Bengani H., Chan C.Y., Kayserili H., Avci Ş. (2014). Genetic Heterogeneity in Cornelia de Lange Syndrome (CdLS) and CdLS-like Phenotypes with Observed and Predicted Levels of Mosaicism. J. Med. Genet..

[47] Ascaso Á., Arnedo M., Puisac B., Latorre-Pellicer A., Del Rincón J., Bueno-Lozano G., Pié J., Ramos F.J. (2024). Cornelia de Lange Spectrum. An. Pediatría (Engl. Ed.).

[48] Olley G., Ansari M., Bengani H., Grimes G.R., Rhodes J., von Kriegsheim A., Blatnik A., Stewart F.J., Wakeling E., Carroll N. (2018). BRD4 Interacts with NIPBL and BRD4 Is Mutated in a Cornelia de Lange-like Syndrome. Nat. Genet..

[49] Jouret G., Heide S., Sorlin A., Faivre L., Chantot-Bastaraud S., Beneteau C., Denis-Musquer M., Turnpenny P.D., Coutton C., Vieville G. (2022). Understanding the New BRD4-Related Syndrome: Clinical and Genomic Delineation with an International Cohort Study. Clin. Genet..

[50] Pié J., Gil-Rodríguez M.C., Ciero M., López-Viñas E., Ribate M.P., Arnedo M., Deardorff M.A., Puisac B., Legarreta J., de Karam J.C. (2010). Mutations and Variants in the Cohesion Factor Genes NIPBL, SMC1A, and SMC3 in a Cohort of 30 Unrelated Patients with Cornelia de Lange Syndrome. Am. J. Med. Genet. A.

[51] Deardorff M.A., Bando M., Nakato R., Watrin E., Itoh T., Minamino M., Saitoh K., Komata M., Katou Y., Clark D. (2012). HDAC8 Mutations in Cornelia de Lange Syndrome Affect the Cohesin Acetylation Cycle. Nature.

[52] Deardorff M.A., Wilde J.J., Albrecht M., Dickinson E., Tennstedt S., Braunholz D., Mönnich M., Yan Y., Xu W., Gil-Rodríguez M.C. (2012). RAD21 Mutations Cause a Human Cohesinopathy. Am. J. Hum. Genet..

[53] Deardorff M.A., Noon S.E., Krantz I.D., Adam M.P., Feldman J., Mirzaa G.M., Pagon R.A., Wallace S.E., Amemiya A. (1993). Cornelia de Lange Syndrome. GeneReviews^®^.

[54] Boyle M.I., Jespersgaard C., Brøndum-Nielsen K., Bisgaard A.-M., Tümer Z. (2015). Cornelia de Lange Syndrome. Clin. Genet..

[55] Infante E., Alkorta-Aranburu G., El-Gharbawy A. (2017). Rare Form of Autosomal Dominant Familial Cornelia de Lange Syndrome Due to a Novel Duplication in SMC3. Clin. Case Rep..

[56] Gervasini C., Picinelli C., Azzollini J., Rusconi D., Masciadri M., Cereda A., Marzocchi C., Zampino G., Selicorni A., Tenconi R. (2013). Genomic Imbalances in Patients with a Clinical Presentation in the Spectrum of Cornelia de Lange Syndrome. BMC Med. Genet..

[57] Rentas S., Rathi K.S., Kaur M., Raman P., Krantz I.D., Sarmady M., Tayoun A.A. (2020). Diagnosing Cornelia de Lange Syndrome and Related Neurodevelopmental Disorders Using RNA Sequencing. Genet. Med..

[58] Bestetti I., Crippa M., Sironi A., Bellini M., Tumiatti F., Ballabio S., Ceriotti F., Memo L., Iascone M., Larizza L. (2024). Long-Read Sequencing Reveals Chromothripsis in a Molecularly Unsolved Case of Cornelia de Lange Syndrome. Front. Genet..

[59] Dey A., Ellenberg J., Farina A., Coleman A.E., Maruyama T., Sciortino S., Lippincott-Schwartz J., Ozato K. (2000). A Bromodomain Protein, MCAP, Associates with Mitotic Chromosomes and Affects G(2)-to-M Transition. Mol. Cell. Biol..

[60] French C.A., Miyoshi I., Aster J.C., Kubonishi I., Kroll T.G., Dal Cin P., Vargas S.O., Perez-Atayde A.R., Fletcher J.A. (2001). BRD4 Bromodomain Gene Rearrangement in Aggressive Carcinoma with Translocation t(15;19). Am. J. Pathol..

[61] You J., Croyle J.L., Nishimura A., Ozato K., Howley P.M. (2004). Interaction of the Bovine Papillomavirus E2 Protein with Brd4 Tethers the Viral DNA to Host Mitotic Chromosomes. Cell.

[62] French C.A., Miyoshi I., Kubonishi I., Grier H.E., Perez-Atayde A.R., Fletcher J.A. (2003). BRD4-NUT Fusion Oncogene: A Novel Mechanism in Aggressive Carcinoma. Cancer Res..

[63] Devaiah B.N., Gegonne A., Singer D.S. (2016). Bromodomain 4: A Cellular Swiss Army Knife. J. Leukoc. Biol..

[64] Drumond-Bock A.L., Bieniasz M. (2021). The Role of Distinct BRD4 Isoforms and Their Contribution to High-Grade Serous Ovarian Carcinoma Pathogenesis. Mol. Cancer.

[65] Lewis M., Wu S.-, Chiang C. (2022). Conditional Human BRD4 Knock-In Transgenic Mouse Genotyping and Protein Isoform Detection. Bio-protocol.

[66] Kanno T., Kanno Y., LeRoy G., Campos E., Sun H.-W., Brooks S.R., Vahedi G., Heightman T.D., Garcia B.A., Reinberg D. (2014). BRD4 Assists Elongation of Both Coding and Enhancer RNAs by Interacting with Acetylated Histones. Nat. Struct. Mol. Biol..

[67] Hnisz D., Abraham B.J., Lee T.I., Lau A., Saint-André V., Sigova A.A., Hoke H.A., Young R.A. (2013). Super-Enhancers in the Control of Cell Identity and Disease. Cell.

[68] Rao S.S.P., Huang S.-C., Glenn St Hilaire B., Engreitz J.M., Perez E.M., Kieffer-Kwon K.-R., Sanborn A.L., Johnstone S.E., Bascom G.D., Bochkov I.D. (2017). Cohesin Loss Eliminates All Loop Domains. Cell.

[69] *Online Mendelian Inheritance in Man, OMIM^®^*; MIM Number: 620568; Johns Hopkins University, Baltimore, MD, USA, 18 December 2023. https://www.omim.org/entry/620568.

[70] Jung M., Philpott M., Müller S., Schulze J., Badock V., Eberspächer U., Moosmayer D., Bader B., Schmees N., Fernández-Montalván A. (2014). Affinity Map of Bromodomain Protein 4 (BRD4) Interactions with the Histone H4 Tail and the Small Molecule Inhibitor JQ1. J. Biol. Chem..

[71] Huang Z., Chu T., Ma W. (2025). Multiple Functions of BRD4 in Maintaining Genomic Stability in Cancers. Biochem. Biophys. Res. Commun..

[72] Alesi V., Dentici M.L., Loddo S., Genovese S., Orlando V., Calacci C., Pompili D., Dallapiccola B., Digilio M.C., Novelli A. (2019). Confirmation of *BRD4* Haploinsufficiency Role in Cornelia de Lange–like Phenotype and Delineation of a 19p13.12p13.11 Gene Contiguous Syndrome. Ann. Hum. Genet..

[73] Ptacek L.J., Opitz J.M., Smith D.W., Gerritsen T., Waisman H.A. (1963). The Cornelia de Lange Syndrome. J. Pediatr..

[74] Jackson L., Kline A.D., Barr M.A., Koch S. (1993). De Lange Syndrome: A Clinical Review of 310 Individuals. Am. J. Med. Genet..

[75] Deardorff M.A., Kaur M., Yaeger D., Rampuria A., Korolev S., Pie J., Gil-Rodríguez C., Arnedo M., Loeys B., Kline A.D. (2007). Mutations in Cohesin Complex Members SMC3 and SMC1A Cause a Mild Variant of Cornelia de Lange Syndrome with Predominant Mental Retardation. Am. J. Hum. Genet..

[76] Rohatgi S., Clark D., Kline A.D., Jackson L.G., Pie J., Siu V., Ramos F.J., Krantz I.D., Deardorff M.A. (2010). Facial Diagnosis of Mild and Variant CdLS: Insights from a Dysmorphologist Survey. Am. J. Med. Genet. A.

